# Mineral soil conditioner requirement and ability to adjust soil acidity

**DOI:** 10.1038/s41598-020-75192-5

**Published:** 2020-10-23

**Authors:** Xiangdong Yang, Yashuang Feng, Xiaohong Zhang, Mingxue Sun, Dan Qiao, Juan Li, Xiaoyan Li

**Affiliations:** 1 Key Laboratory of Plant Nutrition and Fertilizer, Ministry of Agriculture and Rural Affairs/Institute of Agricultural Resources and Regional Planning, Chinese Academy of Agricultural Sciences, Beijing, 100081 China; 2 College of Environmental Sciences, Sichuan Agricultural University, Chengdu Campus, Chengdu, 611130 Sichuan China; 3 Tianjin Cement Industry Design and Research Institute Co., Ltd, Tianjin, 300400 China

**Keywords:** Ecology, Environmental sciences

## Abstract

Mineral soil conditioners (MSCs) are used to regulate soil acidity and improve soil quality; they are often made in sintering potassium feldspar, limestone, or dolomite, and are alkaline materials rich in silicon, calcium, potassium, and magnesium. The key point of how to apply them into farmlands is their ability to adjust soil acidity and the MSCs requirement (MSC_R_). In this study, inductively coupled plasma-optical emission spectroscopy (ICP-OES) analysis and X-ray diffraction (XRD) were firstly used to determine the elemental and phase compositions of the MSCs in order to establish its equivalent relationship for the depletion of soil activity (H^+^) and its conversion relationship with CaCO_3_. Secondly, the soil culture method and the improved Shoemaker Mclean Peatt–Double Buffer (SMP–DB) method were compared using a group of 14 typical acid soils in MSC_R_. It is investigated that the MSCs contained four alkali/alkaline earth–metal elements: Ca, Mg, K, and Na in the bound aluminosilicate form (Ca_2_MgAlSi_2_O_7_, Ca_3_(SiO_3_)_3_, KAlSiO_4_, and KAlSi_2_O_6_); and the depletion of 2.31 mol of H^+^ required 100 g of MSCs and the amount of Si–Ca–K–Mg MSC needed to deplete the same quantity of H^+^ was only 0.87 times that of CaCO_3_. Based on the calculations by using the SMP-DB method and the soil culture method, the MSC_R_ for treating the 14 typical acid soils were in the range of 0.56–8.27 t hm^−2^ and 0–10.8 t hm^−2^, respectively. Data from both methods were highly correlated with each other and there was a good linear correlation between them, and the equation: ${{MSC}_{R}}^{{\prime}}=30.29d-0.77$ could be used to calculate the MSCs requirement. The recommended MSC_R_ was approximately 4–8, 2–6, and 1–3 t hm^−2^ when soil pH < 4.50, 4.50 < pH < 5.50, and pH > 5.50, respectively. The experimental and computational methods established in this study could serve as the scientific basis and theoretical guidance for the production and agricultural use of MSCs.

## Introduction

Soil acidification is relatively slow under natural conditions. However, the process has accelerated under the continuous impact of human activities, which in turn has negative effects on the environment and agricultural production. The acidification of farmland soils has similarly accelerated due to the factors such as the occurrence of acid rain^1^ and excessive use of nitrogen fertilizers^2,3^. The pH of farmland soils in China decreased by approximately 0.5 units during 1980s to 2000s^4^, corresponding to a 2.2-fold increase in soil acidity (H^+^). Soil acidification is usually accompanied by the loss of base cations such as Ca, Mg, K, and Na^5^, dissolution of activated aluminum (Al)^6,7^, and activation of Cd—a toxic heavy metal^8^.

Currently, soil acidification is usually treated with lime, ore powder, plant ash, or organic fertilizers^9,10^. Mineral soil conditioners (MSCs) are alkaline in nature and contain mineral nutrients such as Si, Ca, and K^11^. When MSCs are applied to acid soils, they react with H^+^ to release base cations, thereby improving soil nutrient status and regulating soil pH. As such, they are regarded as good materials for adjusting soils. The raw material for preparing Si–Ca–K–Mg MSCs is K-feldspar, which is sintering with limestone (CaCO_3_) and dolomite (CaMg(CO_3_)_2_)^12,13^. The reaction equations are as follows:

$${\mathrm{KAlSi}}_{3}{\mathrm{O}}_{8}+\mathrm{CaO}\to {\mathrm{KAlSi}}_{2}{\mathrm{O}}_{6}+{\mathrm{CaSiO}}_{3}$$

$${\mathrm{KAlSi}}_{3}{\mathrm{O}}_{8}+2\mathrm{CaO}\to {\mathrm{KAlSiO}}_{4}+{2\mathrm{CaSiO}}_{3}$$

$${\mathrm{Al}}_{2}{\mathrm{O}}_{3}+2\mathrm{CaO}+{\mathrm{SiO}}_{2}\to {\mathrm{Ca}}_{2}{\mathrm{Al}}_{2}{\mathrm{SiO}}_{7}$$

$${2\mathrm{CaSiO}}_{3}+\mathrm{MgO}\to {\mathrm{Ca}}_{2}{\mathrm{MgSi}}_{2}{\mathrm{O}}_{7}$$

The mineral nutrient bioavailability, ability to adjust soil acidity, and the application amount in fields should be investigated firstly if we want to evaluate the performance of a new type of soil conditioner such as MSCs. However, existing studies mainly focus on their effects on crop yield^14,15^ and its role in reducing Cd pollution^16^. And the application amounts recommended by these studies were all determined according to the elemental changes in soils, crop yield and quality by using field experiments. Current research does not take into account the properties of the soil conditioners relating to soil pH; consequently, the recommendations for their use in the fields lack the support of soil nutrient stoichiometric analysis.

The application amount of lime—a type of soil conditioner, can be calculated according to its equivalent relationship with H^+^ that it depletes. Neutralization titration, the culture method and the buffer method are commonly used to calculate this. Generally, the buffer method which is that pH of the soil–buffer mixture is measured and the lime requirement (L_R_) is calculated by conversion, is more widely applied in the routine labs due to its sensitive, convenient, and quick-test. The Woodruff’s buffer method^17,18^, Shoemaker Mclean Peatt–Simple Buffer (SMP–SB) method^19^, Shoemaker Mclean Peatt–Double Buffer (SMP–DB) method^20^, and Mehlich’s buffer method^21,22^ are representative of the buffer method, each with their respective limitations^23,24^. Of these, the improved SMP-DB method proposed by Mclean^25^ has a broader pH and greater sensitivity. It also has the greatest accuracy when calculating L_R_^26^, hence it is widely used currently. By using the L_R_ method, the MSC requirement (MSC_R_) could be quickly ascertained by determining their elemental and phase compositions, to establish the calculation relationships for H^+^ depletion.

In this study, inductively coupled plasma-optical emission spectroscopy (ICP-OES) analysis and X-ray diffraction (XRD) were used to determine the elemental and phase composition of the MSC to derive their equivalent relationships for the depletion of H^+^ and conversion relationships with CaCO_3_ firstly. Next, the soil culture method and the improved SMP-DB method were respectively used to calculate L_R_ for the tested 14 typical acid soils and the actual MSC_R_. The application amount of MSC was then calculated based on the amount of lime used in the SMP-DB method, which was in accordance with the MSC–lime conversion relationship. Last, a correlation analysis was performed between the results of the SMP–DB method and the soil culture method, and thus to establish the equation for calculating the MSC_R_. The purpose of this work was to evaluate the MSCs’ ability to adjust soil acidity, and to establish the experimental method for assessing the appropriate application amount of MSCs into soils. These would provide a scientific basis and theoretical guidance for the production and agricultural use of MSCs.

## Materials and methods

### Reagents and equipment

Para-nitrophenol, analytical grade; triethanolamine, analytical grade; calcium acetate (Ca(CO_2_CH_3_)_2_), analytical grade, China National Pharmaceutical Group Co., Ltd. Potassium chromate (K_2_CrO_4_), analytical grade; hydrochloric acid (HCl), analytical grade, Beijing Chemical Works. Calcium chloride (CaCl_2_·2H_2_O), analytical grade, Shanghai Macklin Biochemical Co., Ltd. Sodium hydroxide (NaOH), analytical grade, Xilong Chemical Industry Incorporated Co., Ltd. pH meter, Thermo Fisher Scientific, USA. ICP-OES 730, Agilent. X ray diffractometer (XRD), Bruker D8, Germany.

Soil conditioner: Developed by Tianjin Cement Industry Design and Research Institute Co., Ltd. Specific preparation method: The raw materials were K-feldspar (produced in Inner Mongolia, with ${\mathrm{KAlSi}}_{3}{\mathrm{O}}_{8}$, ${\mathrm{NaAlSi}}_{3}{\mathrm{O}}_{8}$, and SiO_2_ being the main components), CaCO_3_, and CaMg(CO_3_)_2_, which were crushed and ball-milled in order to get the sizes that could pass through an 80 μm sieve, before being mixed in appropriate ratios. Next, these were sintered by using an alumina crucible placed in a box furnace at 1270 °C for 60 min, then naturally cooled inside, and finally ground to approximately 80 μm to obtain the MSCs.

### Soils samples

14 typical acid soils were listed in Table 1, which are from three provinces in China: Hunan (No. 1–5), Sichuan (No. 6–10), and Guangdong (No. 11–14). Soils were sampled in 0–20 cm, with all vegetation residues in the surface layer removed, then were placed indoors, naturally air-dried, and passed through a 2 mm sieve. A portion of each sample was used for testing and analysis, and the remained was used for the culture test.

**Table 1:** Information of the 14 types of acid soil samples in China.

Soil no.	Sampling location	Latitude and longitude	Use	Soil type
**Sichuan Province**				
1	Wolong Town, Qionglai City	N:30.330° E:103.382°	Tea	Yellow soil
2	Maling Town, Ya'an City	N:30.106° E:103.381°	Tea	Purple soil
3	Jiajiang County, Leshan City	N:29.828° E:103.728°	Tea	Yellow soil
4	Pujiang County, Chengdu City	N:30.209° E:103.207°	Kiwifruit	Yellow soil
5	Heilong Town, Meishan City	N:29.911° E:103.793°	Tangerine	Yellow soil
**Hunan Province**				
6	Yanqiao Town, Huaihua City	N:27.272° E:109.476°	Grape	Red soil
7	Zhijiang County, Huaihua City	N:27.254° E:109.393°	Paddy	Purple soil
8	Lanxi Town, Yiyang City	N:28.322° E:112.274°	Paddy	Yellow clayey paddy soil
9	Xiangyin County, Yueyang City	N:28.726° E:112.857°	Paddy	Paddy soil
10	Huishangang Town, Yiyang City	N:28.153° E:112.113°	Grape	Yellow clayey paddy soil
**Guangdong Province**				
11	Chengtian Town, Shantou City	N:23.193° E:116.496°	Tangerine	Paddy soil
12	Xiyang Town, Meizhou City	N:24.189° E:116.216°	Tea	Yellow soil
13	Zhongluotan Town, Guangzhou City	N:23.394° E:113.427°	Paddy	Paddy soil
14	Ducheng Town, Yunfu City	N:23.267° E:111.496°	Tangerine	Yellow soil

### The evaluation of relationship between L_R_ from schematics of ΔpH and soil acidity by using SMP-DB method

**Figure 1:**
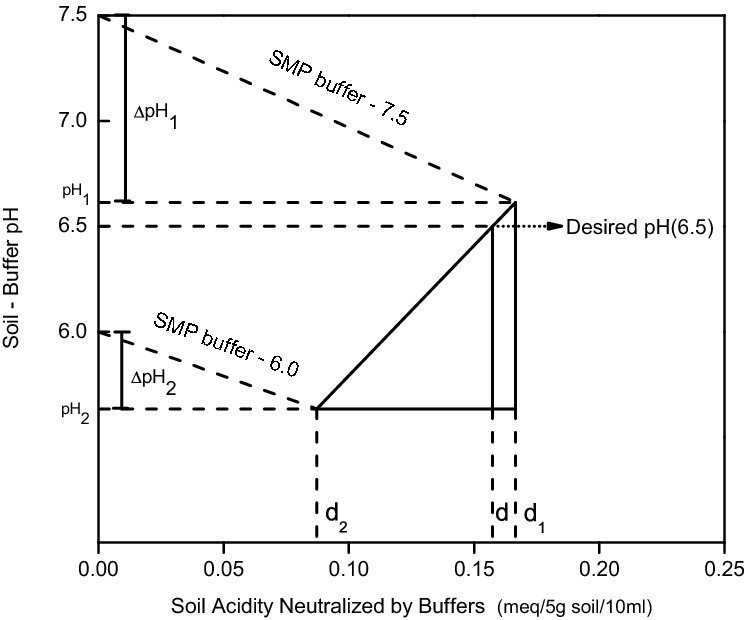
Computation of soil L_R_ from the double buffer schematics and the relationships of the resulting similar triangles. (Notes: *d* = acidity of the soil neutralized by the buffer solution when the soil–buffer solution was at the ideal pH (6.5); *d*_1_ = acidity of the soil neutralized by the buffer solution when pH of the soil–buffer solution reduced from 7.5 to 1; *d*_2_ = acidity of the soil neutralized by the buffer solution when pH of the soil–buffer solution reduced from 6.0 to 2; *pH*_1_ = pH of the soil–buffer solution after addition of the SMP buffer solution; and *pH*_2_ = pH of the soil–buffer solution after addition of HCl.).

Mclean’s improved SMP-DB method was used to calculate L_R_. The calculation principle for the double buffer method is shown in Fig. 1, while the specific operating method and calculation principles of the experiment are as follows.

Based on the schematics of the double buffer method and in accordance with the isosceles triangle principle, the proportional relationship was established as shown in Eq. (1):

1 $$\frac{d-{d}_{2}}{{d}_{1}-{d}_{2}}=\frac{6.5-{pH}_{2}}{{pH}_{1}-{pH}_{2}}$$

Equation (2) was obtained after conversion of Eq. (1):

2 $$d={d}_{2}+\left({d}_{1}-{d}_{2}\right)\frac{6.5-{pH}_{2}}{{pH}_{1}-{pH}_{2}}$$

According to Fig. 1, Eqs. (3) and (4) were obtained by making $\Delta {pH}_{1}=7.5-{pH}_{1}$ and $\Delta {pH}_{2}=6.0-{pH}_{2}$, respectively. ${\left(\frac{\Delta x}{\Delta y}\right)}^{^\circ }$ is the milligram equivalent (meq) of H^+^ that must be depleted to increase the pH of the buffer solution by one unit. It is determined based on the standard curve of the buffer solution.

3 $$\frac{{d}_{1}}{\Delta {pH}_{1}}={\left(\frac{\Delta x}{\Delta y}\right)}^{^\circ }$$

4 $$\frac{{d}_{2}}{\Delta {pH}_{2}}={\left(\frac{\Delta x}{\Delta y}\right)}^{^\circ }$$

Equations (5) and (6) were obtained after conversion of Eqs. (3) and (4):

5 $${d}_{1}=\Delta {pH}_{1}\times {\left(\frac{\Delta x}{\Delta y}\right)}^{^\circ }$$

6 $${d}_{2}=\Delta {pH}_{2}\times {\left(\frac{\Delta x}{\Delta y}\right)}^{^\circ }$$

These were substituted into Eq. (2) and then integrated to obtain Eq. (7):

7 $$\mathrm{d}=\Delta {pH}_{2}\times {\left(\frac{\Delta x}{\Delta y}\right)}^{^\circ }+\left(\Delta {pH}_{1}-\Delta {pH}_{2}\right)\times {\left(\frac{\Delta x}{\Delta y}\right)}^{^\circ }\times \frac{6.5-{pH}_{2}}{{pH}_{1}-{pH}_{2}}$$

Equation (7), intended for theoretical calculations, was derived from the mathematical relationships among the various parameters. $d$ is the equivalent acidity of the soil neutralized by the buffer solution when the soil–buffer solution was at the ideal pH (6.5). The L_R_ required to neutralize 5 g acid soil to pH 6.5 could then be extrapolated based on the measured data and the aforementioned equation.

Since the molecular weight of 1 mol of CaCO_3_ is 100, there is a clear conversion relationship between lime and CaCO_3_. For calculation convenience, L_R_ is commonly expressed as the mass of CaCO_3_ needed to deplete the H^+^ present in 100 g of soil. In other words, ${\mathrm{L}}_{R}= {\text{meq CaCO}}_{3}/100 \,\mathrm{g}=20\mathrm{d}$. After comparing the results obtained via the SMP-DB method and the Ca(OH)_2_-titrated acidity method, Mclean found that Eq. (8), a revision of the earlier equation, had a better correlation with the actual situation.

8 $${\mathrm{L}}_{R}= {\text{meq CaCO}}_{3}/100 \,\mathrm{g}=1.69\left(20d\right)-0.86$$

Under normal circumstances, the weight of a 20 cm thick layer of ploughed soil would be 2250 t per hectare. For one hectare of soil, the L_R_ of CaCO_3_ could be calculated using Eq. (9), with the unit being tons per hectare. This amount is expressed in meq CaCO_3_/100 g soil; to obtain approximate rates in metric tons per hectare (0–20 cm), it can be multiplied by 1.125.

9 $${L}_{R}= {\text{meq CaCO}}_{3}/100 \,\mathrm{g}\times 1.125=\left[1.69\left(20d\right)-0.86\right]\times 1.125=38.03d-0.97$$

### SMP-DB buffer performance

#### Preparation of buffer solution^27^

800 mL distilled water was poured into a 1 L beaker, and then 1.8 g of para-nitrophenol, 2.5 mL of triethanolamine, 3.0 g of K_2_CrO_4_, 2.0 g of Ca(CO_2_CH_3_)_2_, and 53.1 g of CaCl_2_·2H_2_O were added into the beaker; the mixture was then stirred and mixed. NaOH 40% (w/w) or HCl 50% (v/v) was used to adjust the pH to 7.5 before the buffer solution was transferred to a 1 L volumetric flask. The beaker was rinsed for 3 times with 50 mL of distilled water, and the rinses were transferred to the volumetric flask. The eventual constant volume was 1 L.

#### Test method for buffer standard curve titration

1 mL of 0.05 M HCl was added into a 50 mL beaker with 10 mL of buffer solution in it and the pH of the solution was measured after stirring for 1 min. This operation was repeated 8 times and the corresponding pH was recorded to plot a titration curve.

**Figure 2:**
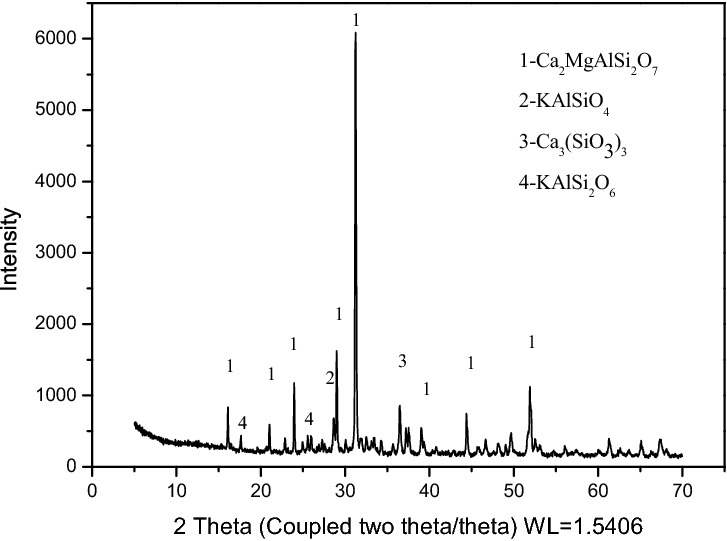
XRD pattern of the MSCs.

The standard curve of the prepared buffer solution is shown in Fig. 2. It has a pH of 5–8 and its standard curve is linear, which could be fitted using a proportional function. The fitting yielded the linear equation y = −7.19x + 8.05, r^2^ = 0.998, which was highly significant. The buffering performance of the buffer solution was calculated based on the fitting equation, and the specific calculation process is stated below.

Two points (*x*_1_, *y*_1_), (*x*_2_, *y*_2_) were selected and substituted into the fitting equation to obtain Eqs. (10) and (11):

10 $${y}_{1}=-7.19{x}_{1}+8.05$$

11 $${y}_{2}=-7.19{x}_{2}+8.05$$

The two equations were subtracted to obtain Eq. (12):

12 $${\mathrm{y}}_{1}-{\mathrm{y}}_{2}=-7.19\left({x}_{1}-{x}_{2}\right)$$

Let $\Delta y={y}_{1}-{y}_{2}, \Delta x=-\left({x}_{1}-{x}_{2}\right)$, then Eq. (13) would be established.

13 $$\frac{\Delta y}{\Delta x}=\frac{{y}_{1}-{y}_{2}}{-\left({x}_{1}-{x}_{2}\right)}=7.19$$

When the pH increased by one unit, ∆ y = 1 was substituted into Eq. (13) to obtain Eq. (14):

14 $${\left(\frac{\Delta x}{\Delta y}\right)}^{^\circ }=\frac{-\left({x}_{1}-{x}_{2}\right)}{{y}_{1}-{y}_{2}}=\frac{1}{7.19}=0.139$$

In other words, 0.139 meq of H^+^ must be depleted per unit increase in the pH of the buffer solution.

During the process of the buffer titration test, the pH of the buffer was adjusted from 7.5 to 6.0. It was shaken again to determine pH_2_. Titration was performed using 0.05 M HCl (1 mL of 0.05 M HCl = 0.05 meq HCl). When the pH of the buffer solution was adjusted from 7.5 to 6.0, the reduction of 1.5 units required 0.139 × 1.5 = 0.2085 meq HCl, which converted to 4.2 mL of 0.05 M HCl.

#### Test procedure for buffer titration^28^

Soil pH was measured using a glass electrode pH meter. 5.00 g soil sample was weighed and placed in a 50 mL beaker. Deionized water was added at a 1:1 water-to-soil ratio, and the beaker was shaken for 10 min at 250 r min^−1^. After standing for 30 min, the pH (suspension) was measured. 10.00 mL of the SMP buffer solution was added and the mixture was shaken again for 10 min and then allowed to stand for 30 min. The suspension’s pH was measured to obtain pH_1_.

After measuring the pH and pH_1_, 4.2 mL of 0.05 M HCl was added to the suspension. This was the equivalent amount needed to adjust the buffer solution’s pH from 7.5 to 6.0 and was calculated according to the buffer solution’s standard curve. The mixture was shaken again for 10 min, and stand for 30 min before the pH of the soil suspension (pH_2_) was measured. The steps were repeated for 3 times.

### ICP-OES measurement

The MSC main elemental contents were determined using ICP-OES. The operating parameters are presented in Table 2.

**Table 2:** Operating parameters of the ICP-OES spectrometer.

Item	Setting
RF power	1.0 kW
Carrier gas	Argon
Plasma flow	15 L min^−1^
Auxiliary flow	1.5 L min^−1^
Nebulizer flow	0.75 L min^−1^
Detector mode	Axial mode
Calibration type	Linear

0.200 g of each MSC was weighed and put into a 30 mL platinum crucible, and 1.500 g of molten agent was added (the mass ratio of sodium carbonate to sodium tetraborate was 2:1). After the molten agent and samples were mixed, the crucible was placed in a muffle furnace and its temperature was raised to 950 °C for 60 min to melt the contents. The crucible was taken out of the furnace after cooling and the sample inside was leached using 70 mL of HCl (3 + 7) to reach a constant volume of 100 mL. This solution was directly used to determine the Ca, Mg, Ba, Ti, and Mn content. Next, the solution was diluted 10 times to determine the high concentrations of K, Al, Si, Na, and Fe content.

### XRD measurement

The MSC samples were ground to 0.045 mm (300 mesh) using an agate mortar and uniformly distributed inside sample frames. These were then pressed, flattened, and compacted using glass slides before being placed on the sample stage of the XRD sample chamber for analysis. The powder XRD patterns were obtained using a Bruker D8 Advance powder diffractometer working at 40 kV and 40 mA, using monochromatized Cu Kα radiation (λ = 0.154056 nm). The measurement was performed in the range angle 2θ = 15°–70°. Before the XRD test, all the samples were ground to 80 μm. The MDI Jade 5.0 software package (USA Materials Data Inc.) was used for qualitative analysis of the XRD spectra being tested.

### Soil culture experiment

The soil samples were mixed with the MSCs and cultured for 30 days. Changes in the soil pH values were used to calculate the MSCs’ pH adjustment capacity and MSC_R_. The test treatments involved the addition of 0, 0.2%, 0.4%, 0.8%, 1.2%, or 1.6% of MSCs (total six levels) to the 14 soil samples and have 3 replications. The specific operating steps were as follows: 50 g of each soil sample and MSCs were mixed in each plastic cup uniformly, and water was added to 60% of the field moisture capacity. The cups were then sealed with plastic film to prevent excessive evaporation. The soil pH was measured after 30 days (1:1 water-to-soil ratio, measured after 10 min of shaking)^29^. The measurements were repeated twice. The measured pH and actual MSC_R_ were subjected to regression analysis, and the regression equation was used to determine the MSC_R_ required to neutralize the soil pH to 6.5 (the ideal pH for this study).

## Results

### Elemental and phase compositions of the MSCs

The soil L_R_ could be calculated according to CaCO_3_ requirement. Since the MSCs were mixtures rich in Ca, K, Mg and other elements, their elemental contents and respective proportions had to be determined firstly, thereby facilitating the theoretical calculations for H^+^ neutralization. ICP-OES was used for elemental analysis of the MSC samples, and the results are shown in Table 3. The SiO_2_ and CaO content was 40.2% and 32.3%, respectively; the MgO, K_2_O, and Na_2_O content was 14.8%, while that of Fe_2_O_3_ and Al_2_O_3_ was 12.1%. The pH of aqueous MSCs was approximately 11, indicating that it was rich in alkaline substances. Assuming that the effect of other metal elements was negligible, the main elements causing the MSCs to be alkaline were four alkali/alkaline earth–metal elements—Ca, Mg, K, and Na.

**Table 3:** Mass percentage and molar amount of elements contained in the MSCs, and molar amount of neutralized H^+^.

Elements composition	SiO_2_	Al_2_O_3_	Fe_2_O_3_	CaO	MgO	K_2_O	Na_2_O	Total
Molecular weight	60.08	101.96	159.69	56.08	40.30	94.20	61.98	–
Mass fraction (%)	40.18	10.54	1.55	32.32	4.80	7.10	2.87	99.36
Mole content* (mol)	0.67	0.10	0.01	0.58	0.12	0.075	0.05	–
H^+^ molar equivalents (mol)	–	0.62	0.06	1.15	0.24	0.15	0.09	2.31

*Molar contents of elements found in 100 g of MSCs.

**Figure 3:**
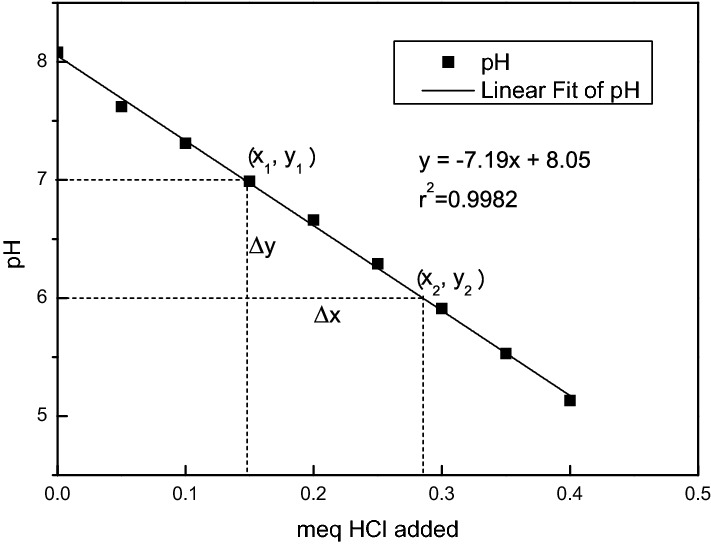
Standard curve of the buffer solution.

The MSCs’ XRD pattern is shown in Fig. 3. The main phases in the K-feldspar MSCs were akermanite-gehlenite (Ca_2_MgAlSi_2_O_7_), pseudowofllastonite (Ca_3_(SiO_3_)_3_), K aluminum silicate (KAlSiO_4_), and Leucite (KAlSi_2_O_6_). The four metal elements Ca, Mg, K, and Na mainly existed in the bound aluminosilicate form.

The decomposition of feldspar-containing K minerals is a hydrolytic process affected by the interface, and the component release conforms to the kinetics of a zero-order reaction^30^. Their hydrolyzability strengthens after sintering, making them easily dissolvable in 2% citric acid^13^. Hence, the MSC_R_ could be calculated based on the total molar number of H^+^ depleted by the metal oxides of Ca, Al, Mg, K, and Na. The molar content of 100 g of the MSCs’ metal elements and the molar number of H^+^ to be depleted were calculated according to the elemental composition determined by ICP-OES and shown in Table 3. The total amount of H^+^ depleted by 100 g of the MSCs was 2.31 mol.

### Quantitative relationship of the MSCs’ ability to adjust soil acidity

After the MSCs were applied to the acid soils, the oxides would react with the soil H^+^ to bring it back to an acid–base equilibrium state. The chemical reaction is expressed using Eq. (15).

15 $${X}_{m}{O}_{n}+2n{H}^{+}=n{H}_{2}O+m{X}^{\frac{2n}{m}+}$$

where *X* is the main metal element in the MSCs (namely Al, Fe, Ca, Mg, K, and Na) and *m* and *n* represent the number of metal and oxygen atoms, respectively.

Using CaCO_3_ as the basis for calculating L_R_, the chemical reaction of CaCO_3_ depleting H^+^ in the soil could be expressed using Eq. (16).

16 $${\mathrm{CaCO}}_{3}+2{\mathrm{H}}^{+}={\mathrm{Ca}}^{2+}+{\mathrm{H}}_{2}\mathrm{O}+{\mathrm{CO}}_{2}$$

Calculating based on Eq. (16), 100 g (1 mol) of CaCO_3_ could deplete 2 mol H^+^. According to Eq. (14) and the ratio of the elemental contents of the MSCs (Table 3), 86.58 g of MSCs was needed to deplete 2 mol of H^+^. Thus, the MSC_R_ to deplete the same amount of H^+^ was only 0.87 times that of CaCO_3_. For the neutralization of H^+^ in 100 g of soil, the quantitative relationship for H^+^ depletion by the MSCs and CaCO_3_ is expressed by Eq. (17):

17 $$\frac{{\mathrm{meq}}_{{MSC}/100\mathrm{g}}}{{\mathrm{meq}}_{{\mathrm{CaCO}}_{3}/100\mathrm{g}}}=\frac{2.00 \mathrm{mol}}{2 .31\mathrm{ mol}}$$

Equation (18) was obtained by rearranging Eq. (17):

18 $${\mathrm{meq}}_{{MSC}/100 \mathrm{g}}=0.8658{\mathrm{meq}}_{{\mathrm{CaCO}}_{3}/100\mathrm{g}}$$

Substituting Eq. (9) into Eq. (18), the MSC_R_ required per hectare of soil was calculated using Eq. (19):

19 $${\mathrm{MSC}}_{R}=\mathrm{meq}\, {\mathrm{CaCO}}_{3}/100\mathrm{g}\times 1.125\times 0.8658=32.92d-0.84$$

### CaCO_3_ application amount and MSC_R_ for the 14 acid soils as determined by SMP-DB

The pH values of the 14 types of acid soils are shown in Table 4 and in the range of 3.37–6.55. Among these, No. 5 soil sample was extremely acid (pH < 4.50), No. 7 were strongly acid (4.50 < pH < 5.50), and No. 2 were acid (pH > 5.50). In general, the soils of all three southern provinces were strongly acid.

**Table 4:** CaCO_3_ amount and MSC_R_ calculated by using the SMP-DB method.

Soil no.	pH	pH_1_	pH_2_	d_1_	d_2_	d	meq CaCO_3_/100 g	L_R_ (t hm^−2^)	MSC_R1_ (t hm^−2^)
1	4.48	6.30	5.53	0.17	0.06	0.19	5.64	6.35	5.49
2*	3.92	5.69	4.47	0.25	0.21	0.28	8.49	9.55	8.27
3	4.70	6.37	5.02	0.16	0.14	0.16	4.54	5.11	4.42
4	4.24	6.39	5.10	0.15	0.13	0.16	4.44	5.00	4.32
5*	4.88	5.99	4.67	0.21	0.19	0.22	6.56	7.38	6.39
6	4.99	6.47	5.25	0.14	0.10	0.14	4.00	4.50	3.90
7*	3.37	6.18	4.85	0.18	0.16	0.19	5.54	6.23	5.40
8	4.96	6.19	4.93	0.18	0.15	0.19	5.57	6.27	5.43
9	5.43	6.52	5.37	0.14	0.09	0.14	3.74	4.21	3.64
10	6.55	7.06	5.85	0.06	0.02	0.04	0.58	0.65	0.56
11	5.51	6.55	5.36	0.13	0.09	0.13	3.52	3.96	3.43
12*	4.52	4.73	4.03	0.39	0.27	0.67	21.72	24.44	21.16
13*	4.23	6.11	4.74	0.19	0.18	0.20	5.86	6.59	5.71
14	5.30	6.75	5.53	0.1	0.06	0.10	2.38	2.68	2.32
15*	4.23	6.00	4.54	0.21	0.20	0.21	6.28	7.07	6.12

For the five acid soil samples marked with an* (Nos. 2, 5, 7, 12, and 14), the *pH*_*2*_ was less than 4.8. According to the SMP-DB method, 4 g of soil should be used for the double buffering experiment of these samples^25^. Correction calculation was also to be performed using the correction equation ${\mathrm{meqCaCO}}_{3}/100\mathrm{ g}=1.69\left(25d\right)-0.86$. $d$ = acidity of the soil neutralized by the buffer solution when the soil–buffer solution was at the ideal pH (6.5); ${d}_{1}$ = acidity of the soil neutralized by the buffer solution when pH of the soil–buffer solution reduced from 7.5 to 1; ${d}_{2}$ = acidity of the soil neutralized by the buffer solution when pH of the soil–buffer solution reduced from 6.0 to 2; ${pH}_{1}$ = pH of the soil–buffer solution after addition of the SMP buffer solution; and ${pH}_{2}$ = pH of the soil–buffer solution after addition of HCl; L_R_ = Lime requirement; MSC_R1_ = Mineral soil conditioners requirement by using the SMP-DB method.

The pH_1_ and pH_2_ of the 14 types of acid soils, which are determined via the buffer titration test procedure, are shown in Table 4. Using the calculation results of the buffer solution’s standard curve, ${\left(\Delta x/\Delta y\right)}^{^\circ }=0.139$ was substituted into Eqs. (5) and (6) to calculate ${d}_{1}$ and ${d}_{2}$. After calculating $d$ using Eq. (7), it was substituted into Eqs. (8) and (9) to calculate the amount of CaCO_3_ depleted by 100 g and per hectare of soil, respectively. The results are shown in the L_R_ column of Table 4. The amounts of CaCO_3_ used by the 14 soils were 0.65–9.55 t hm^−2^.

The MSC_R_ was converted using Eq. (19), which is the stoichiometric equation for the MSCs and CaCO_3_ application amounts, while the CaCO_3_ amount was obtained using the SMP-DB method. The results, shown in the MSC_R_ column of Table 4, indicate that the amounts for the 14 acid soils were in the range of 0.56–8.27 t hm^−2^.

### ***MSC***_***R***_*** determined by the soil culture test***

The MSCs’ actual abilities to adjust soil acidity were determined by using soil culture test, so as to verify the difference between the actual MSC_R_ versus the amount determined by using the SMP-DB method. The results are shown in Table 5.

**Table 5:** Fitting equations and MSC_R_ by using the soil culture method.

Soil no.	MSCs/Soil ratio						Polynomial fitting equation	R^2^	meq MSC/100 g	MSC_R2_ (t hm^−2^)	Ratio of MSC_R1_ to MSC_R2_	MSC_R_' (t hm^−2^)
	0	0.2	0.4	0.8	1.2	1.6						
**1**	4.70	5.89	7.36	7.87	8.13	8.21	y = 4.64 + 8.77x − 7.18x^2^ + 1.73x^3^ + 0.13x^4^	0.932*	0.27	6.08	0.90	5.84
**2**	3.39	5.13	6.30	7.08	7.60	7.79	y = 3.38 + 11.7x − 14.94x^2^ + 9.45x^3^ − 2.26x^4^	0.996**	0.48	10.80	0.80	8.79
**3**	4.68	6.83	7.32	8.08	8.22	8.30	y = 4.73 + 13.1x − 20.46x^2^ + 14.4x^3^ − 3.65x^4^	0.953*	0.18	4.05	1.10	4.70
**4**	3.86	5.81	6.91	7.83	7.98	8.05	y = 3.86 + 12.1x − 13.59x^2^ + 6.89x^3^ − 1.31x^4^	0.999**	0.31	6.98	0.60	4.60
**5**	4.96	6.63	7.37	7.73	8.22	8.17	y = 4.95 + 12.0x − 20.69x^2^ + 16.13x^3^ − 4.44x^4^	0.999*	0.18	4.05	1.60	6.79
**6**	5.16	7.32	7.87	8.33	8.37	8.35	y = 5.20 + 13.9x − 23.14x^2^ + 16.48x^3^ − 4.18x^4^	0.969*	0.11	2.48	1.60	4.14
**7**	4.30	6.27	7.08	7.72	8.04	8.07	y = 4.32 + 13.0x − 19.78x^2^ + 13.95x^3^ − 3.58x^4^	0.997**	0.25	5.63	1.00	5.73
**8**	5.22	7.35	7.58	7.89	7.96	7.85	y = 5.28 + 13.8x − 26.09x^2^ + 20.26x^3^ − 5.46x^4^	0.908*	0.11	2.48	2.20	5.76
**9**	5.64	7.20	7.41	7.81	7.93	8.00	y = 5.68 + 9.80x − 17.31x^2^ + 13.10x^3^ − 3.46x^4^	0.920*	0.10	2.25	1.60	3.87
**10**	6.64	7.84	8.05	8.12	8.11	7.95	y = 6.66 + 8.17x − 15.50x^2^ + 11.95x^3^ − 3.21x^4^	0.951**	0.00	0.00	–	0.60
**11**	5.90	7.49	7.86	8.18	8.24	7.90	y = 5.92 + 10.5x − 18.40x^2^ + 13.97x^3^ − 3.80x^4^	0.964**	0.06	1.35	2.50	3.64
**12**	4.22	6.71	7.16	7.62	7.80	7.85	y = 4.27 + 16.3 × 29.27x^2^ + 22.20x^3^ − 5.87x^4^	0.952*	0.20	4.50	1.30	22.48
**13**	5.74	7.97	8.12	8.11	8.12	7.92	y = 5.79 + 15.2x − 31.04x^2^ + 24.75x^3^ − 6.74x^4^	0.899*	0.05	1.13	2.10	6.07
**14**	4.13	6.37	6.98	7.63	7.98	8.04	y = 4.17 + 14.5x − 24.41x^2^ + 18.32x^3^ − 4.87x^4^	0.980*	0.24	5.40	1.10	2.46

*Means significantly different at the level of 0.05, ** means highly significantly different at the level of 0.01; MSC_R2_ = Mineral soil conditioners requirement by using the soil culture method; Ratio of MSC_R1_ to MSC_R2_ = Ratio of Mineral soil conditioners requirement by using the SMP-DB method to Mineral soil conditioners requirement by using the soil culture method; MSC_R_’ = the modified mineral soil conditioners requirement based on the regression analysis between the SMP-DB method and the soil culture method.

After 30-day’s cultivation, the soil pH value showed an overall and gradual increasing trend as the MSC_R_ slowly rose from 0, 0.2%, 0.4%, 0.8%, 1.2%, to 1.6%. In no addition treatment (addition amount was 0), the pH was roughly equivalent to the initial value (see Tables 5). However, the pH of most soils substantially increased to approximately 8 in the treatment involved the additional amount of 1.6% (converted to 36 t hm^−2^). In general, the pH ​​of the 14 soils increased with rising MSC_R_, and tended towards stability at 1.2% and 1.6%. This was consistent with the findings by Liu^31^ and He^32^ regarding the correlation between pH and CaCO_3_ contents of soils. For each type of soil, the numerical fitting method was used for regression according to the soil pH that corresponded to the MSC_R_. After comparing the fitting results of various models, this study revealed that the quartic model had the most ideal fit. The polynomial equations fitted by soil samples 2, 7, 10, and 11 had extremely significant correlations, while that of the other samples had significant correlations.

When the soil pH was neutralized to 6.5, y = 6.5, the optimum MSC_R_ was obtained using the polynomial fitting equation (Table 5). For the 14 types of soil samples, the optimum MSC_R_ was concentrated within the ranges of 0–3, 3–6, and > 6 t hm^−2^. In practical applications, the MSC_R_ for most of the soils was 0.75–3 t hm^−2^ ^14–16^. In comparison, the MSC_R_ values calculated using the theoretical method were mostly above 3 t hm^−2^.

### Correlation analysis between MSC_R_ determined using the SMP-DB method versus the soil culture method

**Figure 4:**
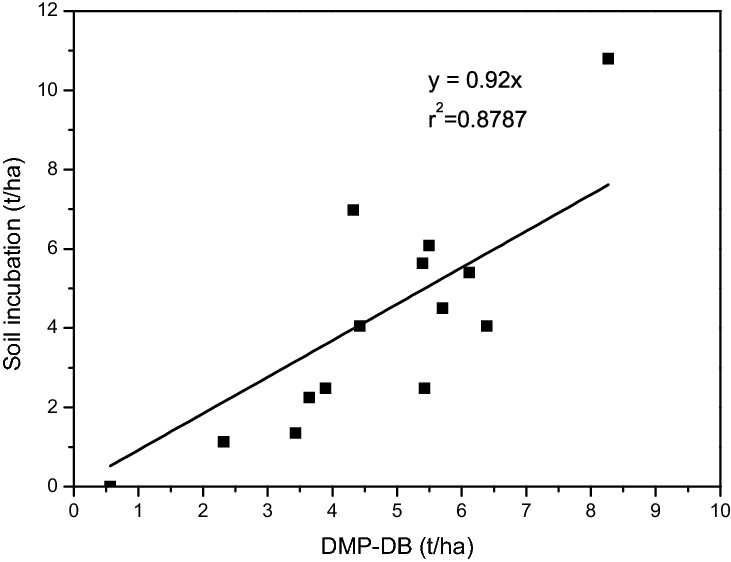
Linear regression curve for the calculated versus actual MSC_R_.

The data of the MSC_R_ determined by the SMP-DB method and the soil culture method (Tables 4 and 5) were similar. For example, the ratios for soil samples NO. 1, 2, 3, 7, and 14 were in the range of 0.8–1.2 (Table 5). However, there were also large differences in some ratios, such as that for samples 8 and 11. These situations might be related to the soil buffer’s performance. The MSC_R_ determined by the SMP-DB method and the soil culture method were subjected to linear regression analysis, and the results are shown in Fig. 4.

The linear regression equation y = 0.92x, r^2^ = 0.879 was obtained by fitting. The CaCO_3_ amount obtained by the soil culture method was used as reference, and Eq. (19) was substituted into the regression equation to obtain the modified ${{MSC}_{R}}^{{\prime}}$ equation based on the SMP-DB method. That equation is shown below:

20 $${{MSC}_{R}}^{{\prime}}=0.92{MSC}_{R}=0.92*{meq}_{MSC/100g}=0.896{meq}_{{\mathrm{CaCO}}_{3}/100g} =30.29d-0.77$$

The MSC_R_' values, according to the modified equation is shown in Table 5, with the amount needed for the 14 soils were found to be in the range of 0.52–7.61 t hm^−2^. Specifically, the amounts for extremely acid soils (pH < 4.50), strongly acid soils (4.50 < pH < 5.50), and acid soils (pH > 5.50) were in the ranges of 3.98–7.61, 2.13–5.88, and 0.52–3.15 t hm^−2^, respectively. After data normalization, the amounts for the three categories of soils were in the ranges of 4–8, 2–6, and 1–3 t hm^−2^.

## Discussion

Zhang^29^ studied 8 types of acid soils from Sichuan Province of China with pH ​​ranging from 4.4 to 5.4 and found that the needed CaCO_3_ amounts were 2–15 t hm^−2^. Yuan^20^ studied 20 types of acid soils in the United States (US) with pH ​​ranging from 3.3 to 6.79 by using the same method and the determined CaCO_3_ amounts were 0.6–11.7 t hm^−2^. Mclean^25^ studied that the pH of 54 types of acid soils in the US was 4.2–6.46 by using the same method and concluded that the required CaCO_3_ amounts were 0.49–12.52 t hm^−2^. All these results are similar to our results, indicating that our results could be used for recommending MSC_R_ to be applied to acid soils.

The modified MSC_R_' ranged from 0.52–7.61 t hm^−2^, whereas the amounts used in the field experiments were generally 0.5–3 t hm^−2^, which were lower than the results obtained in this research. In the previous research, Cao^16^ used Si–Ca–Mg MSCs to restore Cd-contaminated paddy fields in Hunan Province of China and the results showed that 2.25–3 t hm^−2^ was optimal among the 4 treatments of 0, 1.5, 2.25 and 3.0 t hm^−2^ application rates. Li^15^ set up 6 MSC_R_ treatments with 0, 0.6, 0.9, 1.2, 1.5 and 1.8 t hm^−2^ respectively in the water spinach experiments in Fujian Province of China, and they concluded that 1.5 t hm^−2^ was the optimal application amount considering the crop’s yield and economics of production at the same time. Ji^14^ found that the application of 1.5–1.85 t hm^−2^ of Si–Ca–K–Mg MSCs elicited positive results among 5 MSC treatments at 0, 0.75, 1.125, 1.5 and 1.875 t hm^−2^ application amounts in paddy soil in Jiangxi Province of China. Unlike all these previous studies, our study focuses on the ability of MSC to regulate soil acidity, which makes approximately one fold higher application amounts of MSC_R_ than previous results. Furthermore, considering actual production practices, we can control the amount of a once-fertilization within the range of 1 t hm^−2^ per year and apply them over several years to get the soil pH slowly improved. Therefore, the MSC_R_ determined by our proposal method can provide theoretical guidance to apply the MSCs scientifically in the field.

Phase analysis indicated that sintering products of K-feldspar and CaCO_3_ were mainly mixture of silicates and aluminosilicates. Related studies have shown that such sintered products are easily dissolved by 2% citric acid, and that the release of elements such as Si, Ca, K, and Mg of them are enhanced by adjusting the products’ sintering temperature and ingredient ratios^13^. The availability of K and Mg in the MSCs are much higher than those in unsintered K-feldspar. And different ingredients and preparation processes can generate enormous differences in the elemental and phase compositions of MSCs. Whatever, the common goal of producing MSCs is to significantly activate the minerals and enhance the efficiency of elements such as Ca, K, and Mg^33^. Therefore, it was assumed that metal elements could react with H^+^ in the form of metal oxides. The MSCs’ ability to neutralize soil acidity could be calculated based on the number of moles of the various metal elements reacted and the amount of H^+^ depleted in soil. In our study, the MSCs used in the experiment were more alkaline than CaCO_3_ and suitable for adjusting soil acidity based on the results of elemental composition determined by ICP-OES.

**Figure 5:**
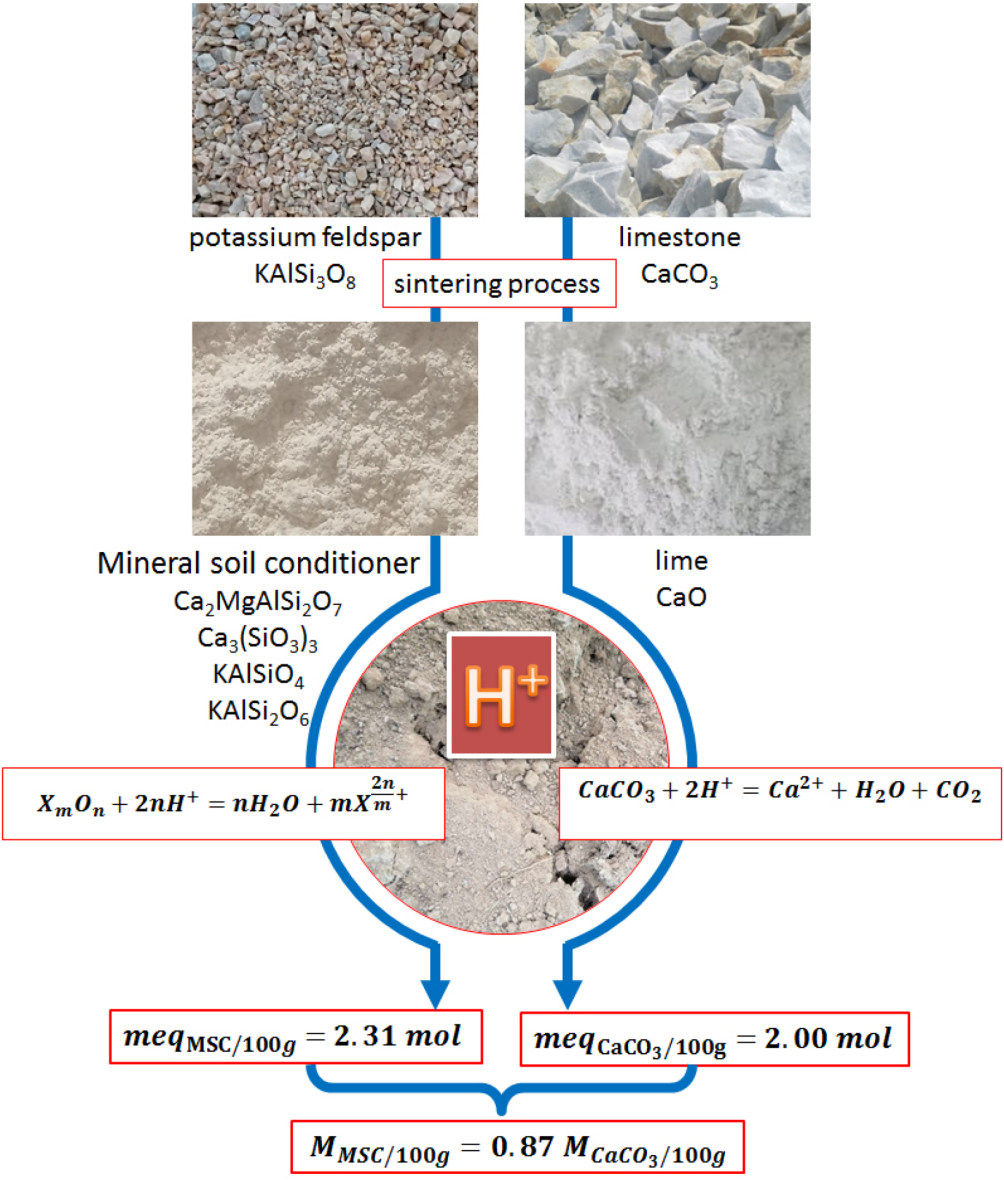
The flow chart of the modified correlations of MSCR between the SMP-DB method and the soil culture method.

The buffering performance of soil pH could be determined either by the SMP-DB method or the soil culture method. The elemental and phase compositions of the MSCs could be used to calculate their ability to neutralize acidity, as well as their conversion relationships with CaCO_3_. Thus, MSC_R_ can be calculated by using the SMP-DB method and the measured results were relatively close to those observed by the soil culture method, meaning that both methods are suitable measures of regulating soil pH (Fig. 5). The modified relationship equation for the MSC_R_, which is based on the SMP-DB method, can provide better guidance for the application of MSCs during production.

The Si– Ca–K–Mg MSCs, formed by sintering K-feldspar and other raw materials, have the same effects as Ca–Mg–phosphate fertilizers. At present, China has a production capacity of approximately 1 million tons of MSCs per year, which is sufficient for applying into 1 million hectares of acid soils based on a once-fertilization of 1 t hm^−2^ practice. In terms of economic value, production–investment balance of MSC_S_ can be achieved if the grain yield increase for 5% in paddy soils according to that the cost of MSCs is approximately 800 yuan per ton. Thus, the proposed MSCs have huge potential to increase economic values for the farmer.

## Conclusions

The MSCs used in this study mainly contain four alkaline earth elements: Ca, Mg, K, and Na, and these elements exist in bound aluminosilicate forms, namely Ca_2_MgAlSi_2_O_7_, Ca_3_(SiO_3_)_3_, KAlSiO_4_, and KAlSi_2_O_6_ by using ICP-OES and XRD analyses. 100 g MSCs was needed to deplete 2.31 mol of H^+^ due to that the SMP-DB buffer solution deplete 0.139 meq of H^+^ per unit increase in pH. And the ability of CaCO_3_ to neutralize soil acidity was only 0.87 times that of the proposed Si–Ca–K–Mg MSCs. The MSC_R_ for the 14 types of acid soils obtained by the SMP-DB method and the soil culture method was measured to be in the range of 0.62–9.13 and 0–10.8 t hm^−2^, respectively. The linear regression equation was found to be y = 0.92x, r^2^ = 0.879, indicating that both methods have a good linear correlation. We determined that the equation ${{MSC}_{R}}^{{\prime}}=30.29d-0.77$ can be used to correct the application amount of MSCs. The recommended MSC_R_ based on the SMP-DB method was approximately 4–8, 2–6, and 1–3 t hm^−2^ when pH < 4.50, 4.50 < pH < 5.50, and pH > 5.50, respectively. The experimental and computational methods established in this study can provide a scientific basis and theoretical guidance for the production and agricultural use of MSCs.

## Data Availability

The data that support the findings of this study are available from the corresponding author, [Li Juan], upon reasonable request.
